# New Metabolites From the Co-culture of Marine-Derived Actinomycete *Streptomyces rochei* MB037 and Fungus *Rhinocladiella similis* 35

**DOI:** 10.3389/fmicb.2019.00915

**Published:** 2019-05-07

**Authors:** Meilin Yu, Yingxin Li, Shivakumar P. Banakar, Lu Liu, Changlun Shao, Zhiyong Li, Changyun Wang

**Affiliations:** ^1^State Key Laboratory of Microbial Metabolism, School of Life Sciences and Biotechnology, Shanghai Jiao Tong University, Shanghai, China; ^2^Key Laboratory of Marine Drugs, The Ministry of Education of China, School of Medicine and Pharmacy, Ocean University of China, Qingdao, China; ^3^Laboratory for Marine Drugs and Bioproducts, Qingdao National Laboratory for Marine Science and Technology, Qingdao, China; ^4^Institute of Evolution and Marine Biodiversity, Ocean University of China, Qingdao, China

**Keywords:** co-culture, actinomycete, fungus, borrelidin, antibacterial activity

## Abstract

Co-culture of different microbes simulating the natural state of microbial community may produce potentially new compounds because of nutrition or space competition. To mine its metabolic potential in depth, co-culture of *Streptomyces rochei* MB037 with a gorgonian-derived fungus *Rhinocladiella similis* 35 was carried out to stimulate the production of new metabolites in this study, using pure cultivation as control. Five metabolites were isolated successfully from co-culture broth, including two new fatty acids with rare nitrile group, borrelidins J and K (**1** and **2**), one chromone derivative as a new natural product, 7-methoxy-2,3-dimethylchromone-4-one (**3**), together with two known 18-membered macrolides, borrelidin (**4**) and borrelidin F (**5**). The structures of **1**–**3** were elucidated by using a combination of NMR and MS spectroscopy, ester hydrolysis, and optical rotation methods. Interestingly, **1** and **2** were obtained only in co-culture. Though **3** was gained from either co-culture or single culture, its production was increased significantly by co-culture. Compound **1** exhibited significant antibacterial activity against methicillin-resistant *Staphylococcus aureus* with a MIC value of 0.195 μg/mL.

## Introduction

In nature, microbes generally exist in a community. One microbe may produce biological products to inhibit other microbes for limited nutrition or space competition or against pathogenic microbes. Thus, co-culture of microorganisms which involves the cultivation of two or more microorganisms in the same confined environment may produce potentially new compounds by stimulating the silent genes or gene clusters of one partner or increase the yields of previously described metabolites. For example, Sung et al. (2017) researched increased production of three antibiotics and enhanced biological activity against the Gram positive human pathogens via co-cultures of a marine-derived *Streptomyces* sp. with human pathogens (Sung et al., 2017), and Zuck et al. (2011) gained a cytotoxic *N*,*N′*-((1*Z*,3*Z*)-1,4-*bis*(4-methoxyphenyl)buta-1,3-diene-2,3-diyl)diformamide by co-culture of the fungus *Aspergillus fumigatus* with the actinomycete *Streptomyces peucetius*.

**FIGURE 1:**
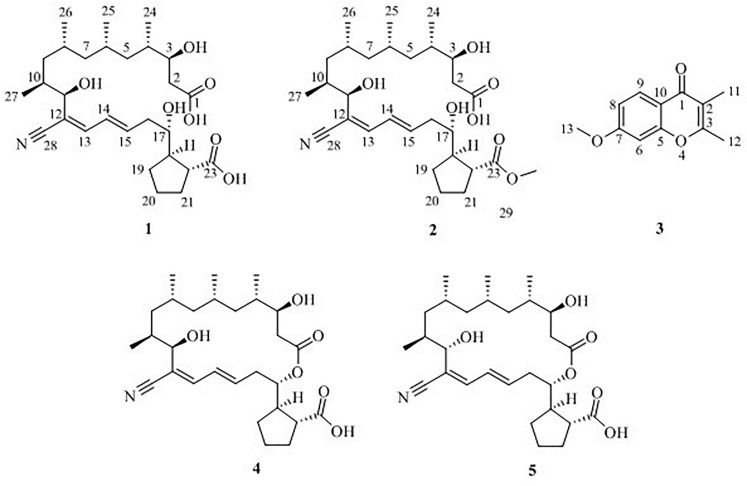
Chemical structures of compounds **1**–**5**.

Macrolide borrelidin has been reported to show broad-spectrum activities (Anderson et al., 1989; Ishiyama et al., 2011; Mirando et al., 2015). In our previous study, borrelidin was isolated and elucidated as a major product from a sponge-derived actinomycete *Streptomyces rochei* MB037 (Li et al., 2018). To mine its more metabolic potential, co-culture approach was applied on *S. rochei* MB037. A gorgonian-derived fungal strain, *Rhinocladiella similis* 35, was selected as a partner against actinomycete *S. rochei* MB037. A literature survey revealed that a few new and bioactive compounds were separated from the fungus *Rhinocladiella* sp. (Wagenaar et al., 2000; Zhang et al., 2014). The co-culture of *S. rochei* MB037 and *R. similis* 35 stimulated the production of new metabolites successfully. Herein, we report the isolation, structural elucidation, and evaluation of biological activities of the metabolites **1**–**5** (Figure 1) produced by co-culture of *S. rochei* MB037 and *R. similis* 35. A plausible biosynthesis pathway for the metabolites was also proposed and discussed.

## Materials and Methods

### Instrumentation

Optical rotations were determined on a JASCO P-2000 polarimeter. 1D and 2D NMR spectra were recorded on an Avance III 600 MHz NMR spectrometer. High-resolution electrospray ionization mass spectroscopy (HR-ESI-MS) was recorded with an ACQUITYTM UPLC and Q-TOF mass spectrometer. High-performance liquid chromatography (HPLC) analysis was performed with an Agilent 1200 detector (G1322A), using a Durashell 150 Å C_18_ column (4.6 mm × 250 mm, 5 μm, Agela) and HPLC preparative-scale purification was performed with an Eclipse XDB C_18_ column (4.6 mm × 150 mm, 5 μm, Agela). The FT-IR spectra were recorded using a Nicolet 6700 spectrometer.

### Microbial Strains

The actinomycete *S. rochei* MB037 was derived from sponge *Dysidea arenaria* collected from Yongxin Island (112° 20′ E; 16° 50′ N) in the South China Sea (Karuppiah et al., 2015). It was identified as *S*. *rochei* on the basis of the 100% 16S rDNA sequence identity with the type strain of this species, under the GenBank Accession No. AA2017041 (Li et al., 2018). The fungal strain *R. similis* 35 was isolated from a staghorn gorgonian collected from Luhuitou fringing reefs (109° 25′ E; 18° 15′ N) in the South China Sea in July 2014. The strain was identified as *R. similis* according to its morphologic traits and molecular identification. Its 616 base pair ITS sequence had 100% sequence identity to that of *R. similis* (KY680425) isolate CMRP1259. The sequence data have been submitted to GenBank, under the Accession No. MH481284. Both of the strains were stored at −80°C after their arrival to the Key Laboratory of Microbial Metabolism, Shanghai Jiao Tong University, China.

### Fermentation, Extraction, and Isolation

The actinomycete *S*. *rochei* MB037 and fungal *R*. *similis* 35 were cultivated in 25 L of ISP2 medium [malt extract 10 g, anhydrous dextrose 4 g, yeast extract 4 g in 1 L of artificial seawater (NaCl 132.6 g, MgCl_2_⋅6H_2_O 55.86 g, CaCl_2_ 5.705 g, KCl 3.625 g, NaHCO_3_ 1.01 g, NaBr 0.415 g), pH value 7.0] at 28°C with shaking at 180 rpm, respectively. On day 3, 200 mL of actinomycete culture was inoculated into each 200 mL of the fungal cultures (1:1 v/v) to initiate the co-culture experiment. The changes of secondary metabolite production between co-culture and single culture were analyzed by reverse-phased HPLC.

After incubation for 11 additional days, the co-culture broth was extracted with 50 L ethyl acetate using rotary evaporator to yield 12 g of reddish brown oil material. The organic extract (12 g) was subjected to Sephadex LH-20 eluting by MeOH to obtain four fractions (Fr. 1–4). Fr. 2–4 was subjected to reversed phase chromatograph eluting with MeOH-H_2_O (Cheetah Fs-9200t C_18_, linear gradient 10, 30, 50, 70, 90, and 100% aqueous CH_3_OH for 30 min, respectively, at a flow rate of 20 mL/min) to give three fractions (Fr. 2–7, 3–18, and 4–8). Fr. 2–7 was further purified twice by preparative HPLC (Eclipse XDB C_18_ column, 4.6 mm × 150 mm, 5 μm, Agela) with 40 to 44% aqueous CH_3_CN to yield **5** (7 mg). Fr. 3–18 was further purified using Sephadex LH-20 with CH_3_OH and then followed by preparative HPLC with 50% aqueous CH_3_CN to give **2** (5 mg), **4** (20 mg) and **5** (4 mg), respectively. Fr. 4–8 was further purified by preparative HPLC with 38% aqueous CH_3_CN to provide **3** (10 mg).

#### Borrelidin J (1)

A light yellow oil; [α]^25^_D_ – 25.5 (*c* 4.0, MeOH); ^1^H and ^13^C NMR spectroscopic data, Tables 1, 2; (−)-HR-ESI-MS m/z 506.3117 [M-H]− (calcd for C_28_H_44_NO_7_, 506.3114).

**TABLE 1:** ^1^H NMR (600 MHz) data for **1**, **2**, **4**, and **5** in pyridine-*d*_5_.

**Position**	**1**		**2**		**4**		**5**	
	***δ*_H_**	**Mult (*J* in Hz)**	***δ*_H_**	**Mult (*J* in Hz)**	***δ*_H_**	**Mult (*J* in Hz)**	***δ*_H_**	**Mult (*J* in Hz)**
2	2.93	m	2.94	m	2.73	m	2.70	m
	2.82	d (13.5)	2.82	m	-	-	2.56	m
3	4.54	m	4.55	m	4.35	m	4.31	d (9.0)
4	1.91	m	2.22	m	1.97	m	2.04	m
5	1.75	m	1.75	m	1.29	m	1.38	m
	1.15	m	1.16	m	0.92	m	0.90	m
6	1.80	m	1.76	m	2.10	m	2.08	m
7	1.27	m	1.28	m	1.02	m	1.11	m
	0.95	m	0.96	m	0.96	m	1.02	m
8	1.75	m	1.78	m	1.72	m	1.57	m
9	1.37	m	1.37	m	1.38	m	1.25	m
	1.23	m	1.23	m	0.94	m	1.05	m
10	2.21	m	1.92	m	2.25	m	2.24	m
11	4.71	d (8.0)	4.71	d (8.0)	4.53	d (8.0)	3.99	d (8.5)
13	6.95	d (11.0)	6.96	d (11.0)	6.82	d (11.0)	6.78	d (11.0)
14	6.85	m	6.84	m	6.65	m	6.69	m
15	6.48	m	6.42	m	6.28	m	6.07	m
16	2.63	m	2.53	m	2.54	m	2.56	m
	2.55	m	2.45	m	2.44	m	2.37	m
17	3.83	m	3.72	m	5.29	m	5.40	m
18	2.75	m	2.62	m	2.84	m	2.82	m
19	1.84	m	1.42	m	1.87	m	1.80	m
	1.48	m	1.25	m	1.25	m	1.29	m
20	1.62	m	1.62	m	1.59	m	1.68	m
21	2.16	m	1.92	m	2.06	m	2.02	m
	2.05	m	1.87	m	1.97	m	1.98	m
22	3.23	m	3.05	dd (8.0, 8.0)	3.04	ddd (8.0, 8.0, 8.0)	2.88	ddd (8.0, 8.0, 8.0)
24	1.11	d (6.5)	1.11	d (6.5)	0.80	d (6.5)	0.78	d (6.5)
25	0.92	d (6.5)	0.93	d (6.5)	0.94	d (6.5)	0.92	d (6.5)
26	0.93	d (6.5)	0.94	d (6.5)	0.87	d (6.5)	0.89	d (6.5)
27	1.27	d (6.5)	1.28	d (6.5)	1.26	d (6.5)	1.22	d (6.5)
29	–	–	3.64	s	–	–	–	–

**TABLE 2:** ^13^C NMR (150 MHz) data for **1**, **2**, **4**, and **5** in pyridine-*d*_5_.

**Position**	**1**		**2**		**4**		**5**	
	***δ*_C_**	**Type**	***δ*_C_**	**Type**	***δ*_C_**	**Type**	***δ*_C_**	**Type**
1	175.4	C	175.3	C	172.1	C	173.0	C
2	41.2	CH_2_	41.1	CH_2_	38.6	CH_2_	38.1	CH_2_
3	70.7	CH	70.7	CH	70.8	CH	70.8	CH
4	36.5	CH	36.5	CH	36.0	CH	35.7	CH
5	41.5	CH_2_	41.5	CH_2_	43.6	CH_2_	43.2	CH_2_
6	27.3	CH	27.3	CH	27.3	CH	26.9	CH
7	45.9	CH_2_	45.9	CH_2_	48.0	CH_2_	48.4	CH_2_
8	27.2	CH	27.2	CH	26.5	CH	26.4	CH
9	39.7	CH_2_	39.8	CH_2_	36.2	CH_2_	37.7	CH_2_
10	35.8	CH	35.8	CH	35.4	CH	35.6	CH
11	71.5	CH	71.5	CH	72.0	CH	78.9	CH
12	117.2	C	120.3	C	118.0	C	117.0	C
13	144.1	CH	144.0	CH	143.4	CH	143.8	CH
14	126.4	CH	126.5	CH	127.5	CH	129.0	CH
15	143.3	CH	143.0	CH	138.7	CH	139.9	CH
16	40.5	CH_2_	40.5	CH_2_	37.7	CH_2_	37.9	CH_2_
17	74.0	CH	73.7	CH	75.8	CH	75.3	CH
18	49.7	CH	50.3	CH	49.4	CH	48.9	CH
19	30.3	CH_2_	29.9	CH_2_	29.6	CH_2_	29.5	CH_2_
20	26.1	CH_2_	25.8	CH_2_	25.4	CH_2_	25.5	CH_2_
21	31.7	CH_2_	31.7	CH_2_	31.6	CH_2_	31.5	CH_2_
22	47.2	CH	46.8	CH	46.2	CH	48.3	CH
23	179.4	C	177.3	C	179.0	C	178.7	C
24	14.7	CH_3_	14.7	CH_3_	18.1	CH_3_	17.9	CH_3_
25	20.0	CH_3_	20.0	CH_3_	18.3	CH_3_	18.4	CH_3_
26	20.9	CH_3_	20.9	CH_3_	20.4	CH_3_	20.2	CH_3_
27	15.4	CH_3_	15.3	CH_3_	15.1	CH_3_	15.8	CH_3_
28	120.4	C	117.3	C	119.9	C	117.4	C
29	–	–	51.3	CH_3_	–	–	–	–

#### Borrelidin K (2)

A light yellow oil; [α]^25^_D_ – 14.0 (*c* 2.5, MeOH); ^1^H and ^13^C NMR spectroscopic data, Tables 1, 2; (−)-HR-ESI-MS m/z 520.3264 [M-H]^–^ (calcd for C_29_H_46_NO_7_, 520.3261).

#### 7-Methoxy-2,3-Dimethylchromone-4-One (3)

A brown yellow solid; ^1^H and ^13^C NMR spectroscopic data, Table 3; (+)-HR-ESI-MS m/z 205.0885 [M+H]^+^(calcd for C_12_H_13_O_3_, 205.0888).

**TABLE 3:** ^1^H (600 MHz) and ^13^C (150 MHz) NMR data for **3** in DMSO-*d*_6_.

**C/H**	**3**			
	***δ*C**	***δ*H**	**Mult (*J* in Hz)**	**HMBC**
1	176.4	–	–	9
2	162.2	–	–	–
3	116.1	–	–	–
5	116.3	–	–	–
6	100.6	7.06	d (2.4)	8, 9
7	163.8	–	–	–
8	114.7	7.01	dd (8.9, 2.4)	6, 9
9	127.0	7.91	d (8.9)	6, 8
10	157.6		–	6, 8, 9
11	10.2	1.93	s	12
12	18.7	2.40	s	–
13	56.5	3.88	s	–

#### Borrelidin (4)

A white amorphous powder; [α]^25^_D_ – 16.0 (*c* 2.5, MeOH); ^1^H and ^13^C NMR spectroscopic data, Tables 1, 2; (−)-HR-ESI-MS m/z 520.3264 [M-H]^–^ (calcd for C_29_H_46_NO_7_, 520.3261).

#### Borrelidin F (5)

A white amorphous powder; [α]^25^_D_ + 14.0 (*c* 2.5, MeOH); ^1^H and ^13^C NMR spectroscopic data, Tables 1, 2; (−)-HR-ESI-MS m/z 520.3264 [M-H]^–^ (calcd for C_29_H_46_NO_7_, 520.3261).

### Antibacterial Bioassay

Antibacterial activity was evaluated by the conventional broth dilution assay (Appendino et al., 2008). Five bacterial strains: *Escherichia coli*, *Pseudomonas aeruginosa*, *Staphylococcus aureus*, *Bacillus subtilis*, and *Bacillus mycoides* were used, and ciprofloxacin was used as a positive control.

## Results

### Co-culture of *S. rochei* MB037 and *R. similis* 35

**FIGURE 2:**
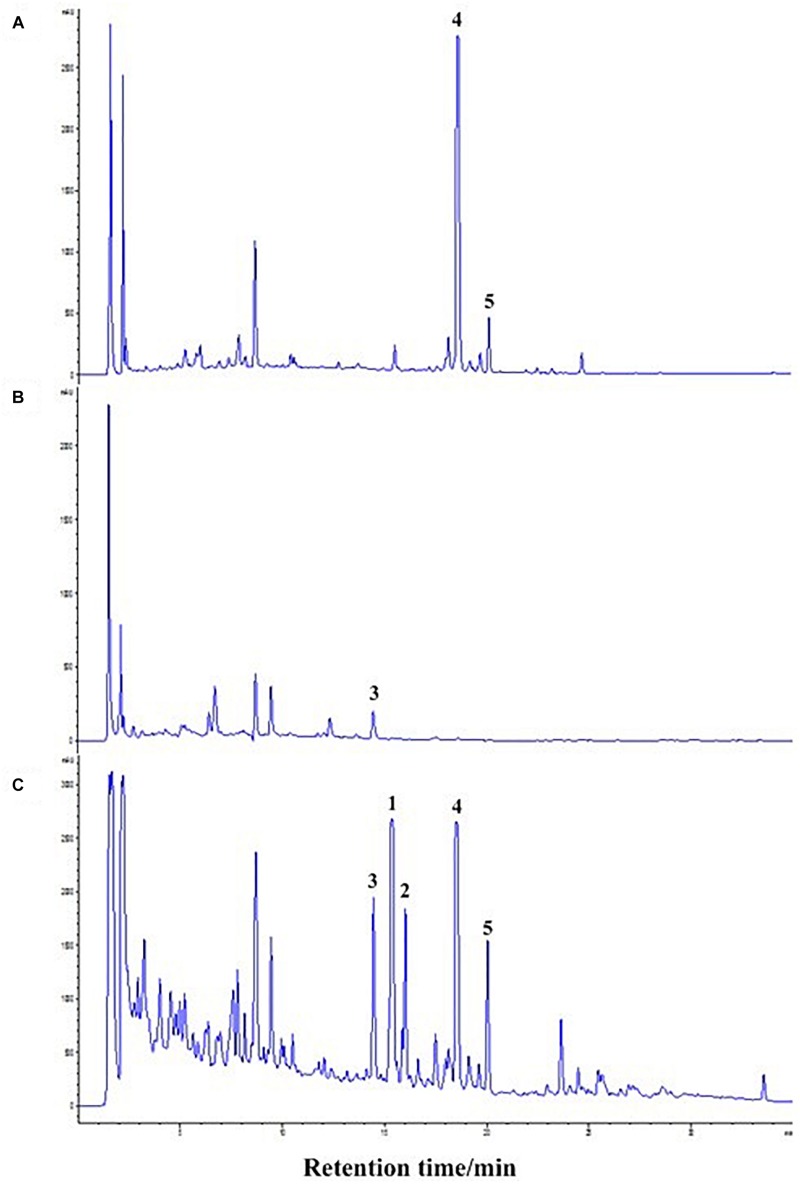
High-performance liquid chromatography (HPLC) profiles of the EtOAc extracts of different culture approaches. **(A)** Pure culture of *Streptomyces rochei* MB037; **(B)** pure culture of *Rhinocladiella similis* 35; **(C)** co-culture of *S*. *rochei* MB037 and *R*. *similis* 35.

**FIGURE 3:**
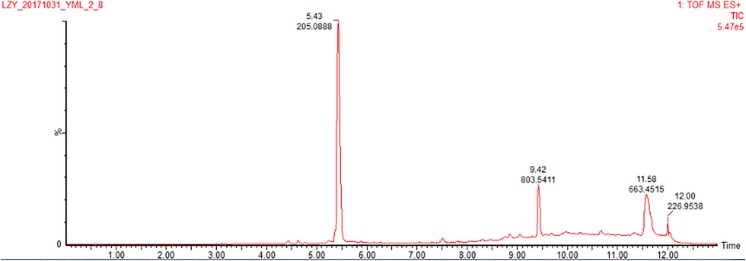
HR-ESI-MS of the EtOAc extracts (positive ion mode) of pure culture of *R*. *similis* 35.

The co-culture of sponge-derived actinomycete *S. rochei* MB037 with multiple marine microorganisms (bacteria and fungi) was tested. Based on a series of screening of co-cultivation with single strain cultivation as control, significant changes in metabolites in the fermentation broth were observed in the co-culture of a gorgonian-derived fungal strain, *R. similis* 35 with *S. rochei* MB037. The actinomycete stain *S*. *rochei* MB037 and fungal strain *R*. *similis* 35 were cultured for 3 days, respectively, and then co-cultivated for 11 days. As a control, the single cultivation was carried out for 14 days. Then the EtOAc extracts of fermentation broth were compared by HPLC (Figure 2). In single strain cultivation, two peaks (**4** and **5**) appeared for *S*. *rochei* MB037 (Figure 2A); a weak peak (**3**) was detected in *R*. *similis* 35 (Figure 2B). Compared with the control, two extra peaks (**1** and **2**) and one obvious increased peak (**3**) were detected in the co-cultural EtOAc extracts (Figure 2C). HR-ESI-MS analysis confirmed that peaks **1** and **2** were not detected in single cultural broth of actinomycete *S*. *rochei* MB037, while peak **3** with the molecular weight 204 ([M + H]^+^ *m/z* at 205.0885) was detected in the single cultural broth of fungal strain *R*. *similis* 35 (Figure 3). This result indicated that the fungus *R. similis* 35 successfully induced the actinomycete *S. rochei* MB037 to produce new metabolites.

Five compounds (**1–5**) (Figure 1) were isolated from the co-culture broth of *S. rochei* MB037 and *R. similis* 35 using column chromatography and preparative HPLC. Besides the known macrolide borrelidin (**4**) and borrelidin F (**5**), *S*. *rochei* MB037 was induced to produce two new fatty acids, borrelidins J (**1**) and K (**2**). Correspondingly, *R*. *similis* 35 was also stimulated to enhance the production of a chromone derivative 7-methoxy-2,3-dimethylchromone-4-one (**3**), which has never been reported as a natural product.

### Structure Elucidation

Borrelidin J (**1**) was isolated as a yellow oil. Its molecular formula C_28_H_45_NO_7_ was confirmed by negative ion HR-ESI-MS ([M – H]^–^ calcd. 506.3117, C_28_H_44_NO_7_^–^) (Supplementary Figure S1) with seven degrees of unsaturation. The IR spectra showed absorption at 3375, 2210, and 1637 cm^–1^, suggesting the presence of hydroxyl, nitrile group, and double-bond protons, respectively. The NMR of **1** (Supplementary Figures S2–S5) closely resembled that of the known compound borrelidin (**4**) expect for the differences at C-1 and C-17 proton signals. The C-1 proton signal at *δ*_C_ 172.1 in **4** was shifted to *δ*_C_ 175.4 in **1**. The H-17 proton signal at *δ*_H_ 5.29 (1H, d, *J* = 10.0 Hz) in **4** was shifted to *δ*_H_ 3.83 (1H, m) in **1**, and this change was consistent with the difference between **4** and **1** of ^13^C signals, the C-17 at *δ*_C_ 75.8 for **4** was shifted to *δ*_C_ 74.0 for **1** on the ^13^C NMR (Table 2 and Supplementary Figure S4). Also, on the HMBC (Supplementary Figure S6), C-1 was related to H-17 in **4**, however, there was no correlation between them in **1**. Besides, compound **4** has one less unsaturation and one more H_2_O on the formula and molecular weight than **1**. On the basis of these data (Supplementary Figures S1–S9), we postulated that **1** was the ester hydrolysis product of **4**.

**FIGURE 4:**
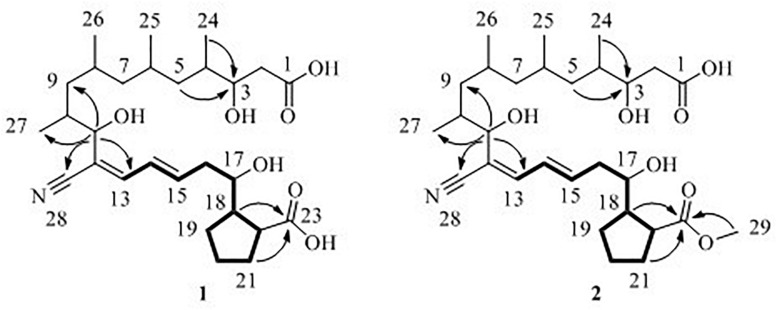
^1^H–^1^H COSY (**—**), key HMBC (↷) correlations of compounds **1** and **2**.

Borrelidin K (**2**) was purified as a yellow oil and its molecular formula was determined to be C_29_H_47_NO_7_ based on the analysis of negative ion HR-ESI-MS ([M – H]^–^ calcd. 520.3264, C_29_H_46_NO_7_^–^) (Supplementary Figure S10). The NMR data (Supplementary Figures S11–S14) of **2** were analogous to those of **1** (Tables 1, 2), except for a carbon signal at *δ*_C_ 51.3 (Table 2 and Supplementary Figure S13), presumed to be an oxygen carbon signal. HSQC (Supplementary Figure S15) correlation of H-29 (*δ*_H_ 3.64, 3H) to C-29 (*δ*_C_ 51.3) and HMBC correlation of H-29 (*δ*_H_ 3.64, 3H) to C-23 (*δ*_C_ 177.3) further confirmed the existence of methoxy group of C-29 (Figure 4 and Supplementary Figure S16). Combined with the relevant information of COSY (Figure 4 and Supplementary Figure S17) and NOSEY spectra (Supplementary Figure S18), the structure of 2 was determined. Therefore, it was confirmed that the methyl group replaced the H atom of the C-23 carboxylic acid in compound **1** to form a methyl ester.

**FIGURE 5:**
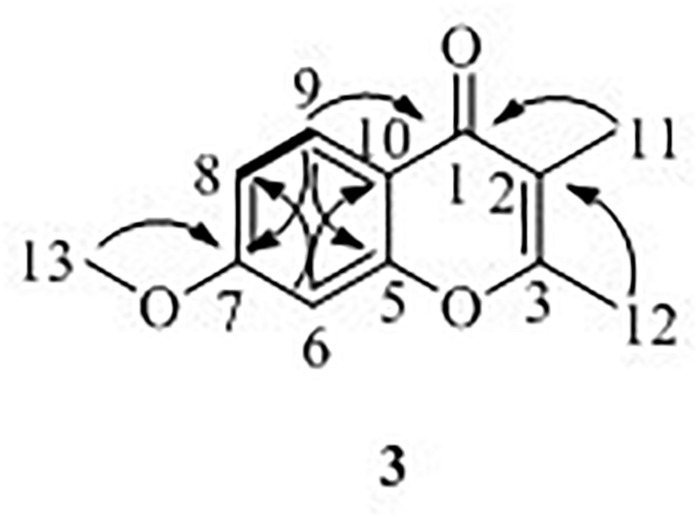
^1^H–^1^H COSY (**—**), key HMBC (**↷**) correlations of compound **3**.

7-Methoxy-2,3-dimethylchromone-4-one (**3**) was isolated as a brown yellow powder. Its molecular formula was deduced as C_12_H_12_O_3_ on the basis of (+)-HR-ESI-MS analysis ([M + H]^+^ calcd. 205.0885, C_12_H_13_O_3_^+^) (Supplementary Figure S19). The ^1^H NMR (Table 3 and Supplementary Figure S20) of **5** in DMSO-*d*_6_ showed three aromatic hydrogens including H-6 (*δ*_H_ 7.06, d, *J* = 2.4 Hz), H-8 (*δ*_H_ 7.01, dd, *J* = 8.9, 2.4 Hz) and H-9 (*δ*_H_ 7.91, d, *J* = 8.9 Hz), and three methyl protons including H-11 (*δ*_H_ 1.93, s), H-12 (*δ*_H_ 2.40, s), and H-13 (*δ*_H_ 3.88, s). ^13^C NMR data showed (Table 3 and Supplementary Figures S21, S22) six quaternary carbons, including one carbonyl carbons C-1 (*δ*_C_ 176.4), two double-bond carbons C-2 (*δ*_C_ 162.2), and C-3 (*δ*_C_ 116.2), and three aromatic carbons C-7 (*δ*_C_ 163.8), C-10 (*δ*_C_ 157.6), and C-5 (*δ*_C_ 116.3). Moreover, the ^13^C NMR data revealed the presence of one methoxy group C-13 (*δ*_C_ 56.5) and three aromatic tertiary carbons including C-9 (*δ*_C_ 127.0), C-8 (*δ*_C_ 114.7), and C-6 (*δ*_C_ 100.6). Correlations of H-9/C-1, C-6, C-7, C-8, and C-10, H-8/C-5, C-6, and C-10, H-13/C-7, and H-11/C-1 were observed in HMBC spectrum (Figure 5 and Supplementary Figure S23). Supported by other NMR data showed in Table 3, and relevant HSQC (Supplementary Figure S24) and COSY (Supplementary Figure S25) spectra data, the structure of **3** was elucidated as a new natural product, 7-methoxy-2,3-dimethylchromone-4-one.

Compound **4** was definitively identified as previously reported borrelidin on the basis of HR-ESI-MS, ^1^H NMR, ^13^C NMR, optical rotation and comparisons with previously reported data (Liu et al., 2012).

Borrelidin F (**5**) was also analyzed and identified comparing with the data of HR-ESI-MS, ^1^H NMR, ^13^C NMR and optical rotation provided in the reported literature (Sun et al., 2018).

### Antimicrobial Screening

The antibacterial activities assay revealed that compounds **1** and **2** exhibited potent activity against methicillin-resistant *S. aureus* with the MICs of 0.195 and 1.563 μg/mL, respectively (Table 4). Compound **1** showed stronger activity than ciprofloxacin while **4** and **5** were inactive, indicating that the cleavage of the ester bond of the macrolides enhanced the antibacterial activity. It could be supposed that co-culture activated the silencing metabolic potential of the actinomycete and produced more potent metabolites of antibacterial activity against microorganism, such as fungal attack.

**TABLE 4:** Antibacterial activities of **1**–**5** (MIC, μg/ml).

	**1**	**2**	**3**	**4**	**5**	**Ciprofloxacin**
*E. coli*	>100	>100	>100	25	>100	0.156
*P. aeruginosa*	>100	50	25	>100	50	0.078
*S. aureus*	0.195	1.563	25	>100	>100	0.313
*B. subtilis*	>100	>100	>100	0.195	12.5	0.039
*B. mycoides*	>100	>100	>100	12.5	50	0.039

### Formation Mechanism Inference

**FIGURE 6:**
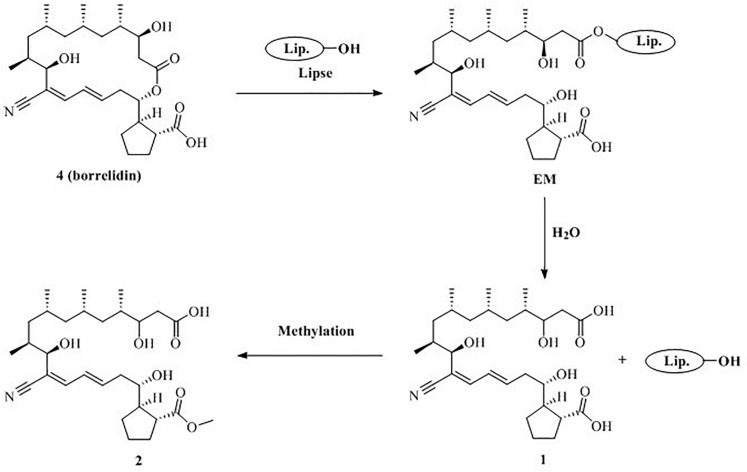
Proposed formation mechanism for **1** and **2**.

Based on the structural characteristics of **1**, **2** and **4**, combined with literature reports (Uyama et al., 1995; Kobayashi, 2006), we

proposed a plausible biosynthesis pathway for new compounds **1** and **2** (Figure 6). Compound **4** may be hydrolyzed to produce **1** by the lipase catalyst due to the mutual stimulation-inducing effect during the co-culture process of the actinomycete *S*. *rochei* MB037 and the fungus *R*. *similis* 35. Meanwhile, compound **2** was obtained when the C-23 carboxyl group of **1** was

methyl esterification. Consequently, it could be concluded that compounds **1** and **2** were derived from the actinomycete *S*. *rochei* MB037.

**FIGURE 7:**
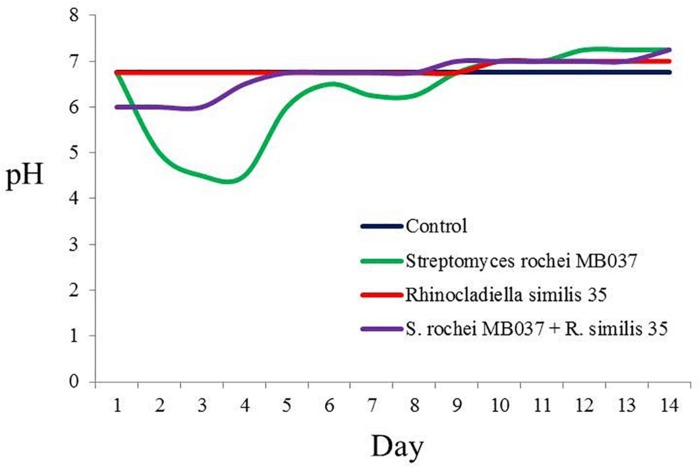
pH trends of pure culture and co-culture of *S*. *rochei* MB037 and *R*. *similis* 35.

**FIGURE 8:**
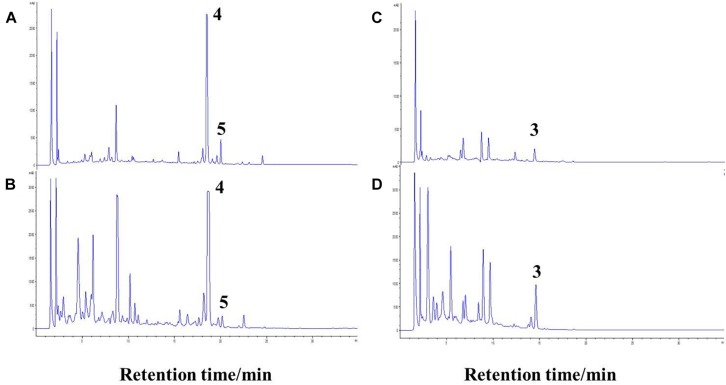
Comparison of pure culture and co-culture. **(A)** Culture of *S*. *rochei* MB037; **(B)** co-culture of *S*. *rochei* MB037 and *R*. *similis* 35; **(C)** culture of *R*. *similis* 35; **(D)** co-culture of *R*. *similis* 35 and *S*. *rochei* MB037.

To investigate the cause for the enhancement of the production of compound **3**, we measured the pH value during the growths of pure and co-cultivation firstly. It was found that the pH value of the pure culture medium of the actinomycete began to decrease on the 2nd day (Figure 7), indicating that the actinomycete began to produce secondary metabolites. Subsequently, the fungus and actinomycete were cultured separately for 3 days and then sterilized. The inactivated cells of fungus or actinomycete were co-cultured with actinomycete or fungus for 3 days (Figure 8). As a result, it was found that when the fungus *R. similis* 35 was co-cultured with the sterilized actinomycete *S. rochei* MB037, compound **3** was still obtained with higher yield than pure culture (Figure 8D). It could be speculated that the secondary metabolites from actinomycete *S*. *rochei* MB037 stimulated the fungus *R*. *similis* 35 to produce **1**.

## Discussion

The fungus *R. similis* 35 was selected as the co-culture partner with *S. rochei* MB037 due to the stable emerging peaks in HPLC profiles of the co-cultures broth. 7-methoxy-2,3-dimethylchromone-4-one (**3**) showed a weak antibacterial activity against *P. aeruginosa* and *S. aureus*, therefore, the increased yield of compound **3** by fungus *R. similis* 35 may aim to inhibit the growth of *S. rochei* MB037. It is common for strains to compete with each other to obtain yield-increasing products in co-culture (Zuck et al., 2011; Sung et al., 2017). Co-culture is an effective method for inducing novel secondary metabolites from two interacted microbial strains. Recent studies revealed that many novel natural products were produced only from the interaction of two microbes (Scherlach and Hertweck, 2009; Hoshino et al., 2015; Wu et al., 2015). For example, the co-culture of two plant beneficial microbes *Trichoderma harzianum* M10 and *Talaromyces pinophilus* F36CF produced a novel harziaphilic acid (Vinale et al., 2017). From the perspective of microbial ecology, it is hypothesized that the production of borrelidin derivatives including two new compounds (**1** and **2**) and the increased yield of 7-methoxy-2,3-dimethylchromone-4-one (**3**) may be caused by the mutual competition for nutrition or space in the co-culture of these two strains.

Genomic sequencing has demonstrated that a large number of putative biosynthetic gene clusters encoding for secondary metabolites in many microorganisms are silent under classical cultivation conditions (Nett et al., 2009; Winter et al., 2011). A distinct fungal-bacterial interaction leads to the specific activation of fungal secondary metabolism genes, which has been demonstrated at the molecular level by microarray analyses, full-genome arrays, Northern blot, and quantitative RT-PCR analyses (Schroeckh et al., 2009). *Rhodococcus erythropolis* and *Corynebacterium glutamicum* were proved to change the biosynthesis of *Streptomyces* to produce new secondary metabolites (Onaka et al., 2011). Studies using chemical inhibitors disclosed that the activity of chromatin remodelers was the main factor for the interaction between *S. rapamycinicus* and *A. nidulans* to produce extra products (Nützmann et al., 2011). We speculated that compounds **1** and **2** were synthesized by the same biosynthetic gene clusters responsible for the biosynthesis of borrelidin according to the structure similarity. Compounds **1** and **2** were only produced by *S*. *rochei* MB037 in co-culture condition indicating that the silent hydrolytic enzyme genes for hydrolyzing lactone in borrelidin could be activated by the co-culture of actinomycete *S. rochei* MB037 and fungus *R. similis* 35. The interaction between these two strains probably activate the expression of hydrolytic enzyme genes, harbored in actinomycete *S. rochei* MB037, and then led to hydrolytic action of lactone of borrelidin to generate compound **1**. Compound **2** is probably synthesized from compound **1** by methylation reaction since the only difference between **1** and **2** is the methyl ester group. During the extraction and purification processes, both of them were clearly detected in freshly prepared ethyl acetate extracts. Indeed, even if compound **1** was dissolved in methanol and stored at 28°C for 1 week, compound **2** was not detected in the solution. Therefore, compound **2** should be considered as a true natural product.

In the antibacterial bioassay, both compounds **1** and **2** exhibited stronger activities against *S. aureus* than **4** and **5**. Notably, the MIC value of compound **1** was 0.195 μg/ml, stronger than the positive control ciprofloxacin, indicating that **1** should be a potential antibacterial agent. It seems that the cleavage of the ester bond of the macrolide in borrelidin could enhance its antibacterial activity. Compounds **1** and **2** become the long-chain unsaturated fatty acid after the cleavage of the ester bond in borrelidin. Previous studies indicated that long-chain unsaturated fatty acid could exhibit strong activity against *S. aureus* by inhibiting the enoyl-acyl carrier protein reductase (FabI), which was the essential component in bacterial fatty acid synthesis (Zheng et al., 2005). However, the esterification of unsaturated fatty acid results in the loss of FabI-inhibitory activity, which is consistent with our results since compounds **4** and **5** exhibited no activity. The antibacterial activity of unsaturated fatty acid was very weak to the Gram negative bacteria due to the impermeability of their outer membrane. Consistently, compounds **1** and **2** showed weak antibacterial activity against *E. coli* and *P. aeruginosa*. Although *Bacillus subtilis* was the Gram positive bacteria, it has two kinds of enoyl-acyl carrier protein reductases, FabI and FabL, which may escape the inhibition of unsaturated fatty acid by alternative enoyl-acyl carrier protein reductase in fatty acid synthesis (Kim et al., 2011). The cytotoxicity of these compounds was not conducted, but their analogs exhibited cytotoxicity to mammalian cells (Zheng et al., 2005; Wilkinson et al., 2006; Chen et al., 2012; Sun et al., 2018).

## Conclusion

This study demonstrated that microbial co-culture was an effective approach to explore the natural products. Based on a series of screening of co-cultivation of marine-derived microbes, a co-cultured combination of a sponge-derived actinomycete *S. rochei* MB037 and a gorgonian-derived fungus *R. similis* 35 was proved to induce the production of related polyketides with antibacterial activities successfully. The two new metabolites (**1** and **2**) produced by co-culture of marine-derived actinomycete and fungus represent the nitrogen-containing fatty acids which are rare in the nature. Future investigation should be focused on unveiling the mechanisms of action in molecular biology of the new produced compounds.

## Author Contributions

MY performed the experiments, data analyses, and wrote the draft manuscript. YL, LL, and CS assisted the bioactivity analysis and revised the manuscript. SB revised the manuscript. ZL and CW supervised the whole work and edited the manuscript. All authors reviewed and approved the final manuscript.

## Conflict of Interest Statement

The authors declare that the research was conducted in the absence of any commercial or financial relationships that could be construed as a potential conflict of interest.
