# Supplementary material for: New Metabolites From the Co-culture of Marine-Derived Actinomycete Streptomyces rochei MB037 and Fungus Rhinocladiella similis 35
Source: Front Microbiol. 2019 May 7;10:915. doi: 10.3389/fmicb.2019.00915 (PMC6514141; doi:10.3389/fmicb.2019.00915)
Supplement: Supplementary file 1 [file Data_Sheet_1.docx]

**Supplemental Material**

**New Metabolites from the Co-culture of Marine-derived Actinomycete *Streptomyces rochei* MB037 and Fungus *Rhinocladiella similis* 35**

Meilin Yu ^1,2,3^, Yingxin Li ^1^, Shivakumar P. Banakar ^1^, Lu Liu ^2,3^, Changlun Shao ^2,3^, Zhiyong Li^*1^, Changyun Wang^*2,3,4^

*^1^State Key Laboratory of Microbial Metabolism, School of Life Sciences and Biotechnology, Shanghai Jiao Tong University, Shanghai 201100, People’s Republic of China.*

*^2^Key Laboratory of Marine Drugs, the Ministry of Education of China, School of Medicine and Pharmacy,* *Ocean University of China, Qingdao 266003, People’s Republic of China.*

*^3^Laboratory for Marine Drugs and Bioproducts, Qingdao National Laboratory for Marine Science and Technology, Qingdao 266200, People’s Republic of China.*

*^4^Institute of Evolution & Marine Biodiversity, Ocean University of China, Qingdao 266003, People’s Republic of China*

Correspondence

Prof. Dr. Zhi-Yong Li

School of Life Sciences & Biotechnology, Shanghai Jiao Tong University; State Key Laboratory of Microbial Metabolism, 800 Dongchuan Road, Minhang District, Shanghai, 200240, P.R.China

Tel: +86-21-34204036

Fax: +86-21-34204036

E-mail: zyli@sjtu.edu.cn

Prof. Dr. Chang-Yun Wang

School of Medicine and Pharmacy, Ocean University of China; Key Laboratory of Marine Drugs, the Ministry of Education, 5 Yushan Road, Qingdao, 266003, Shandong, P. R. China.

Tel: +86/532/82031536

Fax: +86/532/82031536

E-mail: changyun@ouc.edu.cn

_____________________________________________________________________

*Corresponding authors. Email: [zyli@sjtu.edu.cn](mailto:zyli@sjtu.edu.cn); changyun@ouc.edu.cn

## Abstract

In our previous study, a sponge-derived actinomycete *Streptomyces rochei* MB037 was found to produce borrelidin. To mine its metabolic potential, co-culture of *S. rochei* MB037 with a gorgonian-derived fungus *Rhinocladiella similis* 35 was carried out to stimulate the production of new metabolites compared with single strain’s cultivation in this study. From the co-culture broth, five metabolites were isolated successfully, including two new fatty acids with rare nitrile group, borrelidins J and K (**1** and **2**), one chromone derivative as a new natural product, 7-methoxy-2,3-dimethylchromone-4-one (**3**), together with two known 18-membered macrolides, borrelidin (**4**) and borrelidin F (**5**). The structures of **1**–**3** were elucidated by using a combination of NMR and MS spectroscopy, ester hydrolysis, and optical rotation methods. Interestingly, **1** and **2** were obtained only through co-culture, but **3** was gained through either co-culture or single culture, while the production of which was increased significantly by co-culture. Compound **1** exhibited significant antibacterial activity against methicillin-resistant *Staphylococcus aureus* with a MIC value of 0.195 *µ*g/mL.

**Keywords**: co-culture, actinomycete, fungus, borrelidin, antibacterial activity

## List of supporting information

**Figure S1.** HR-ESI-MS spectrum of compound **1**

**Figure S2.** ^1^H NMR (600 MHz, pyridine-*d*_5_) spectrum of compound **1**

**Figure S3**. Partial ^1^H NMR (600 MHz, pyridine-*d*_5_) spectrum of compound **1**

**Figure S4.** ^13^C NMR (150 MHz, pyridine-*d*_5_) spectrum of compound **1**

**Figure S5.** DEPT (150 MHz, pyridine-*d*_5_) spectrum of compound **1**

**Figure S6.** HMBC (pyridine-*d*_5_) spectrum of compound **1**

**Figure S7.** HSQC (pyridine-*d*_5_) spectrum of compound **1**

**Figure S8.** ^1^H–^1^H COSY (pyridine-*d*_5_) spectrum of compound **1**

**Figure S9.** NOSEY (pyridine-*d*_5_) spectrum of compound **1**

**Figure S10.** HR-ESI-MS spectrum of compound **2**

**Figure S11.** ^1^H NMR (600 MHz, pyridine-*d*_5_) spectrum of compound **2**

**Figure S12.** Partial ^1^H NMR (600 MHz, pyridine-*d*_5_) spectrum of compound **2**

**Figure S13.** ^13^C NMR (150 MHz, pyridine-*d*_5_) spectrum of compound **2**

**Figure S14.** DEPT (150 MHz, pyridine-*d*_5_) spectrum of compound **2**

**Figure S15.** HSQC (pyridine-*d*_5_) spectrum of compound **2**

**Figure S16.** HMBC (pyridine-*d*_5_) spectrum of compound **2**

**Figure S17.** ^1^H–^1^H COSY (pyridine-*d*_5_) spectrum of compound **2**

**Figure S18.** NOSEY (pyridine-*d*_5_) spectrum of compound **2**

**Figure S19.** HR-ESI-MS spectrum of compound **3**

**Figure S20.** ^1^H NMR (600 MHz, DMSO-*d*_6_) spectrum of compound **3**

**Figure S21.** ^13^C NMR (150 MHz, DMSO-*d*_6_) spectrum of compound **3**

**Figure S22.** DEPT (150 MHz, DMSO-*d*_6_) spectrum of compound **3**

**Figure S23.** HMBC (DMSO-*d*_6_) spectrum of compound **3**

**Figure S24.** HSQC (DMSO-*d*_6_) spectrum of compound **3**

**Figure S25.** ^1^H–^1^H COSY (DMSO-*d*_6_) spectrum of compound **3**

**New compound statistics**


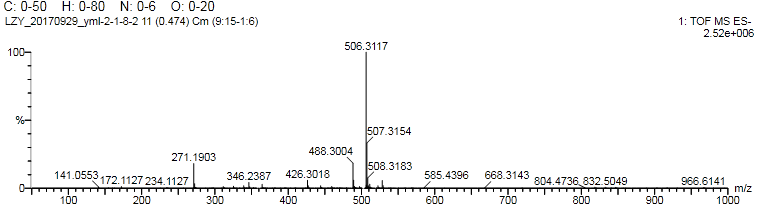


**Figure S1**. HR-ESI-MS spectrum of compound **1**


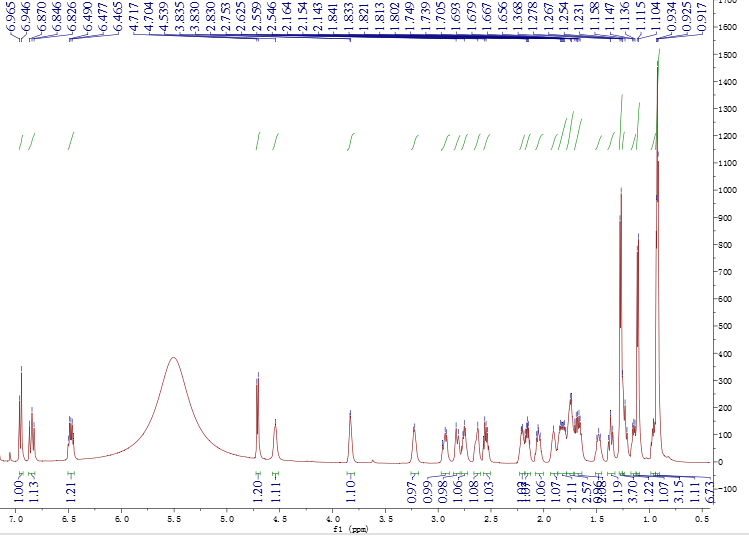


**Figure S2**. ^1^H NMR (600 MHz, pyridine-*d*_5_) spectrum of compound **1**


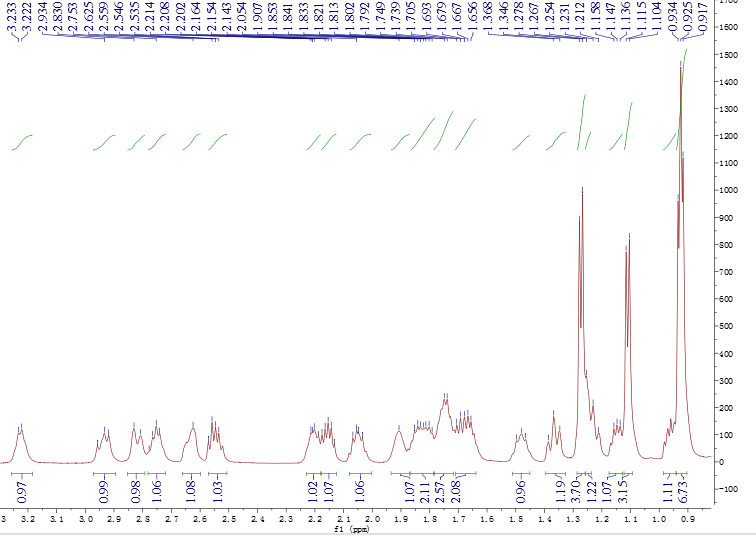


**Figure S3**. Partial ^1^H NMR (600 MHz, pyridine-*d*_5_) spectrum of compound **1**


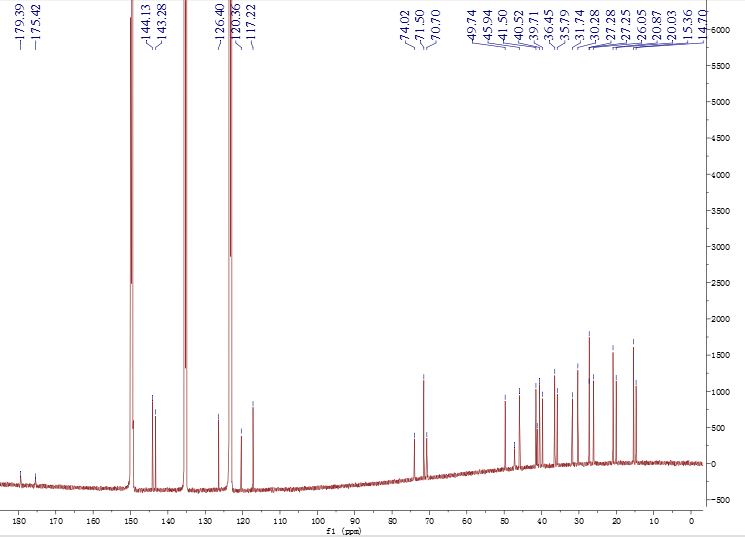


**Figure S4**. ^13^C NMR (150 MHz, pyridine-*d*_5_) spectrum of compound **1**


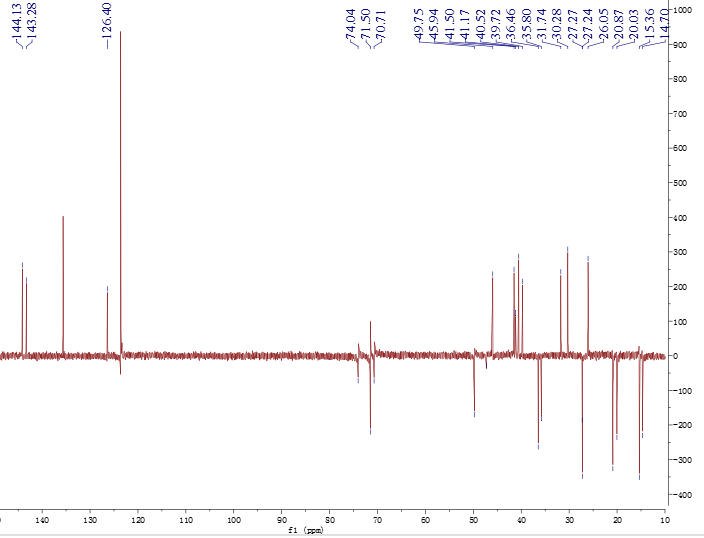


**Figure S5**. DEPT (150 MHz, pyridine-*d*_5_) spectrum of compound **1**
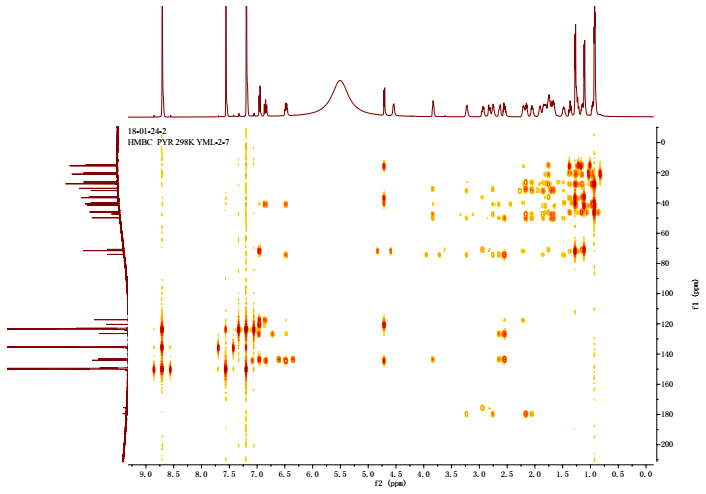


**Figure S6**. HMBC (pyridine-*d*_5_) spectrum of compound **1**


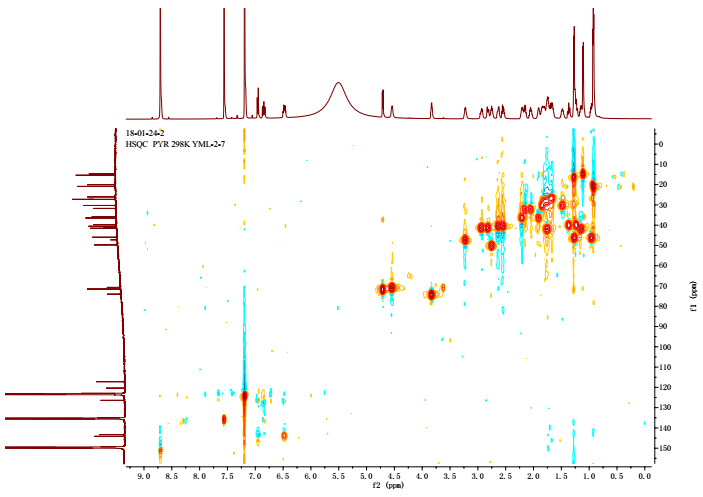


**Figure S7**. HSQC (pyridine-*d*_5_) spectrum of compound **1**


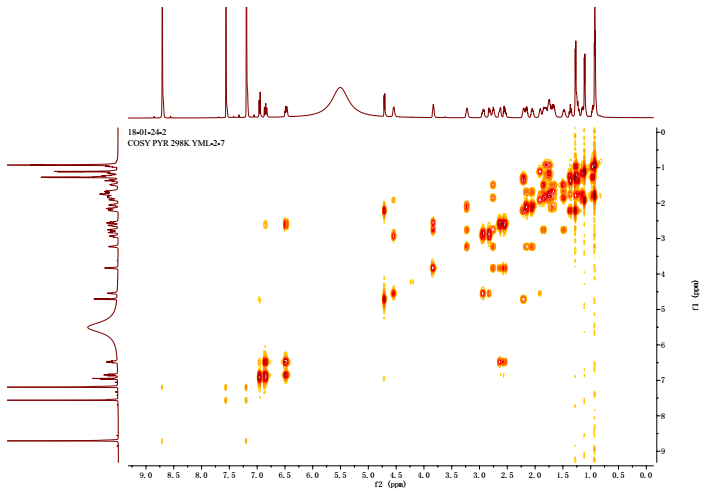


**Figure S8**. ^1^H–^1^H COSY (pyridine-*d*_5_) spectrum of compound **1**


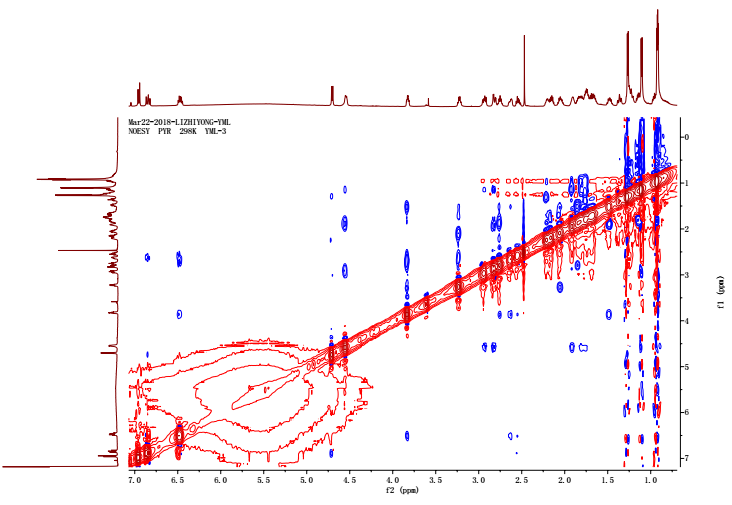


**Figure S9**. NOSEY (pyridine-*d*_5_) spectrum of compound **1**


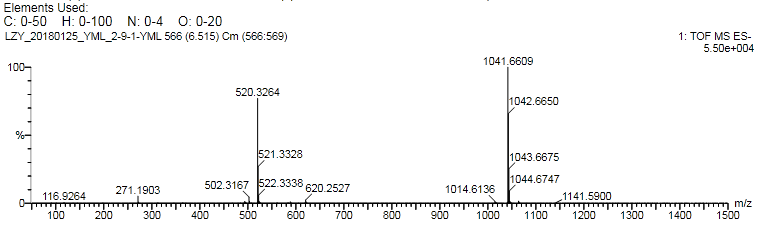


**Figure S10**. HR-ESI-MS spectrum of compound **2**


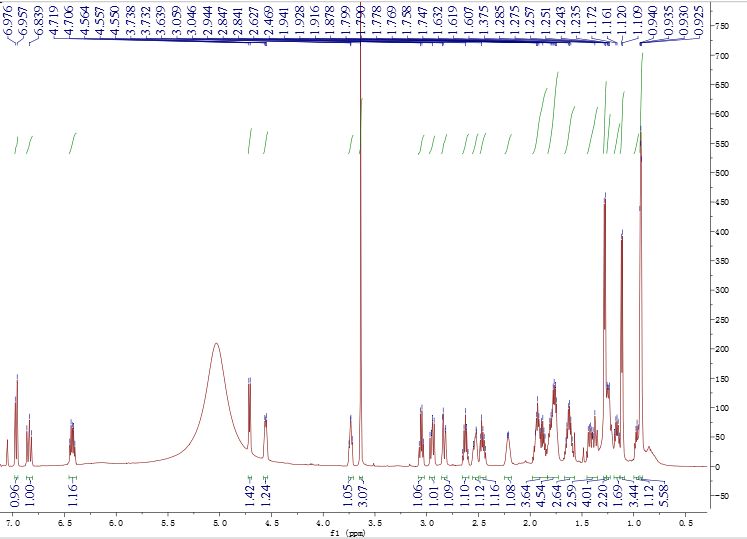


**Figure S11.** ^1^H NMR (600 MHz, pyridine-*d*_5_) spectrum of compound **2**


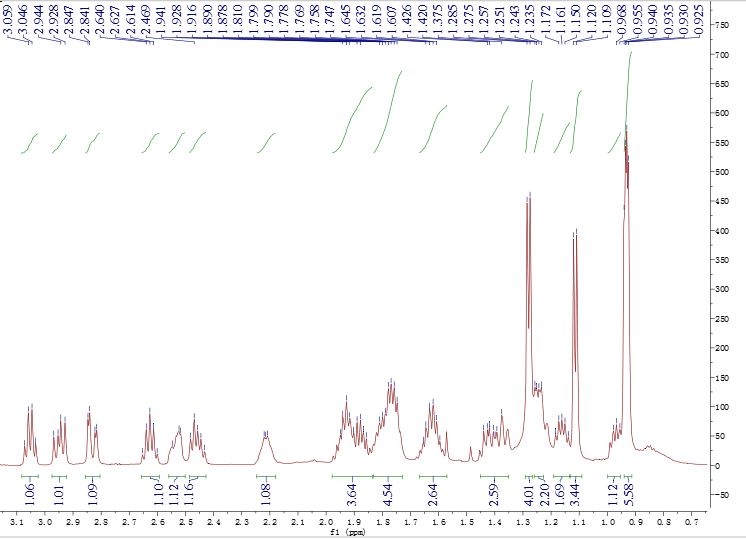


**Figure S12.** Partial ^1^H NMR (600 MHz, pyridine-*d*_5_) spectrum of compound **2**


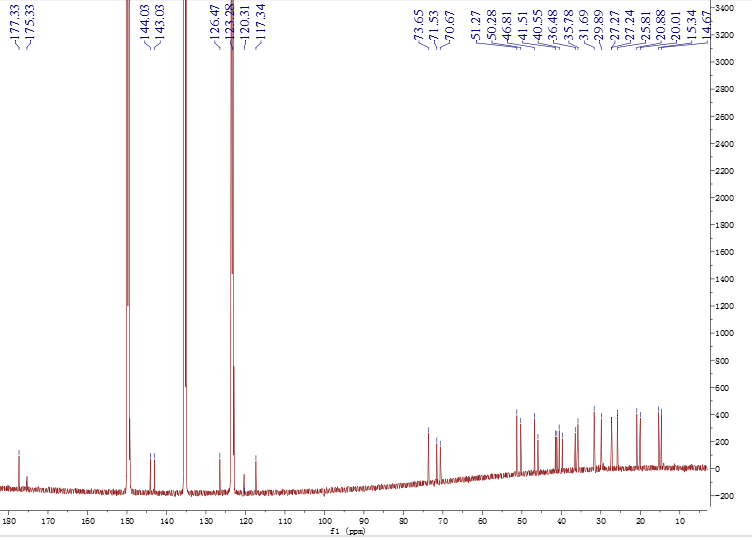


**Figure S13**. ^13^C NMR (150 MHz, pyridine-*d*_5_) spectrum of compound **2**


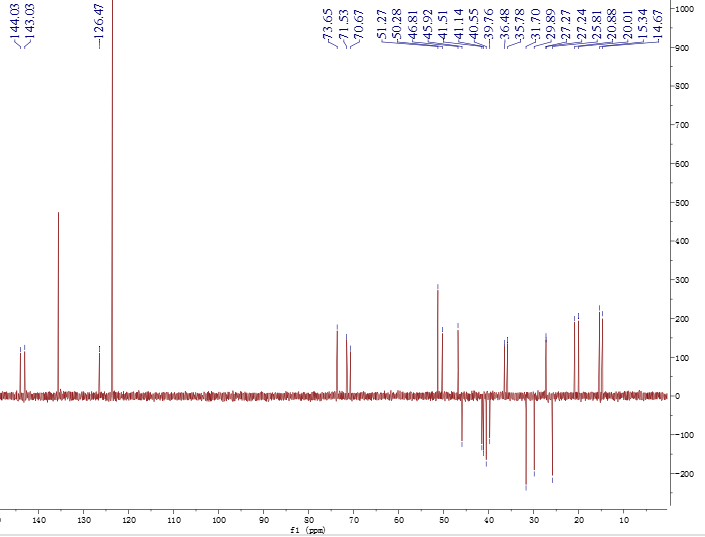


**Figure S14**. DEPT (150 MHz, pyridine-*d*_5_) spectrum of compound **2**


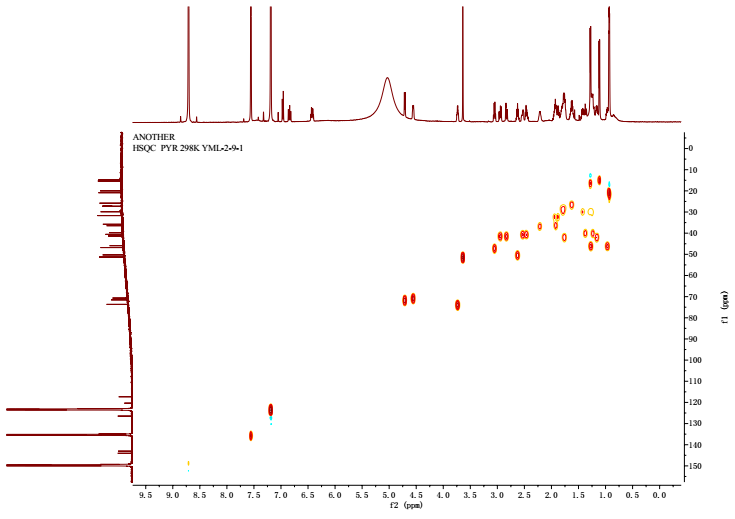


**Figure S15**. HSQC (pyridine-*d*_5_) spectrum of compound **2**
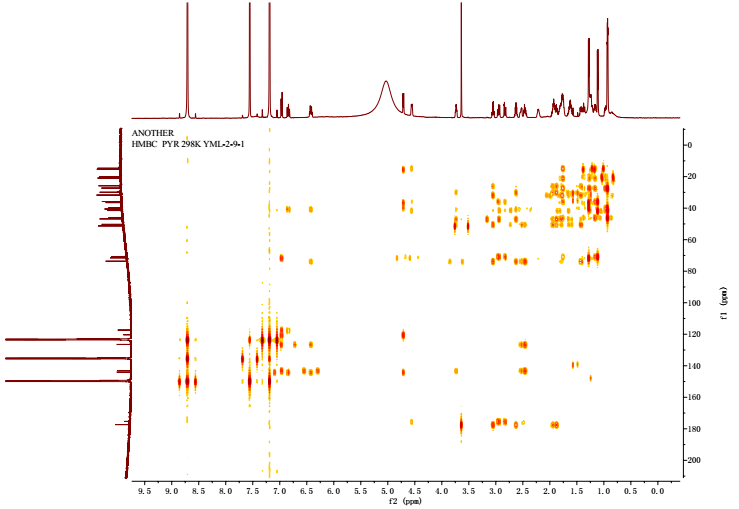


**Figure S16**. HMBC (pyridine-*d*_5_) spectrum of compound **2**


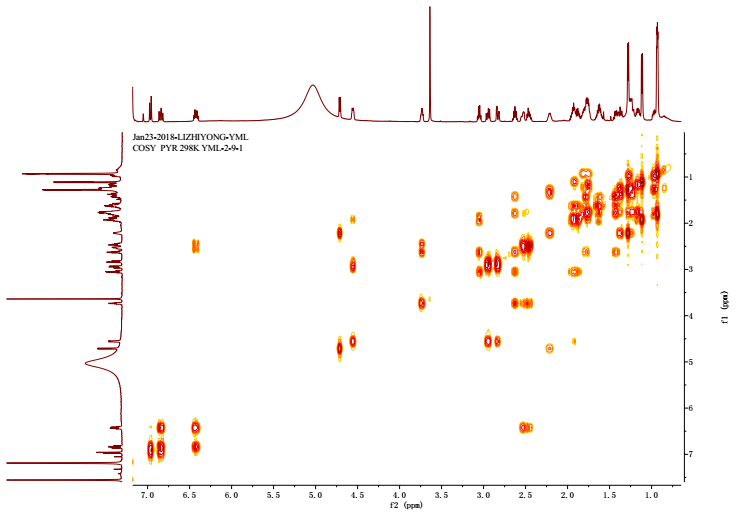


**Figure S17**. ^1^H–^1^H COSY (pyridine-*d*_5_) spectrum of compound **2**


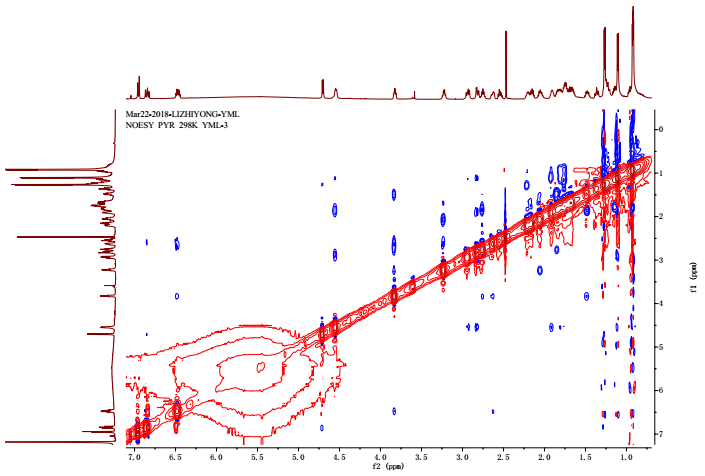


**Figure S18**. NOSEY (pyridine-*d*_5_) spectrum of compound **2**
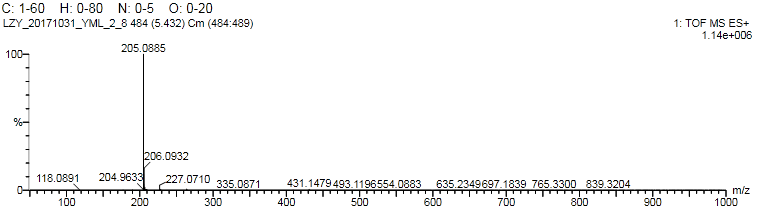


**Figure S19**. HR-ESI-MS spectrum of compound **3**


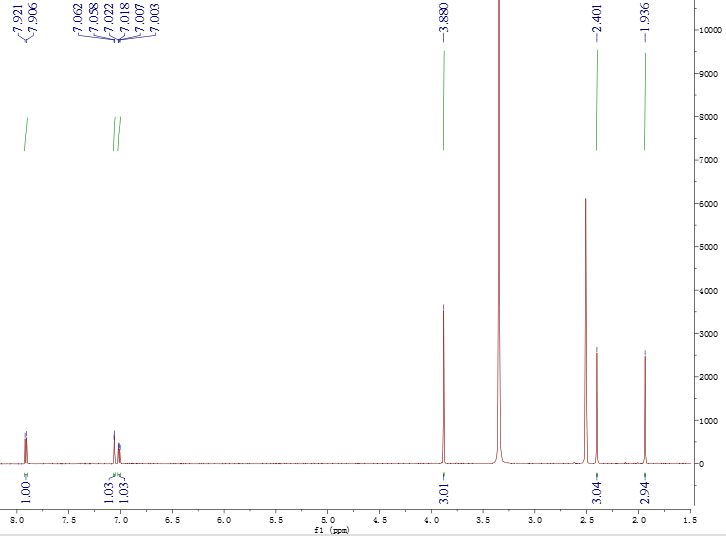


**Figure S20**. ^1^H NMR (600 MHz, DMSO-*d*_6_) spectrum of compound **3**


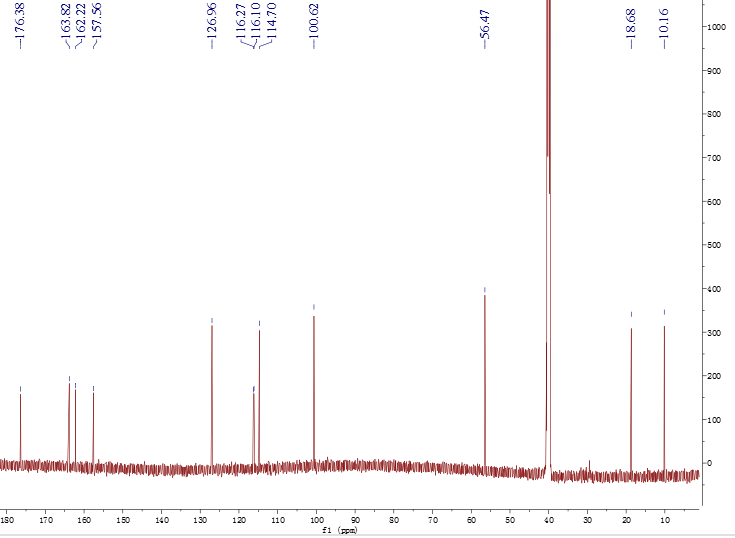


**Figure S21**. ^13^C NMR (150 MHz, DMSO-*d*_6_) spectrum of compound **3**


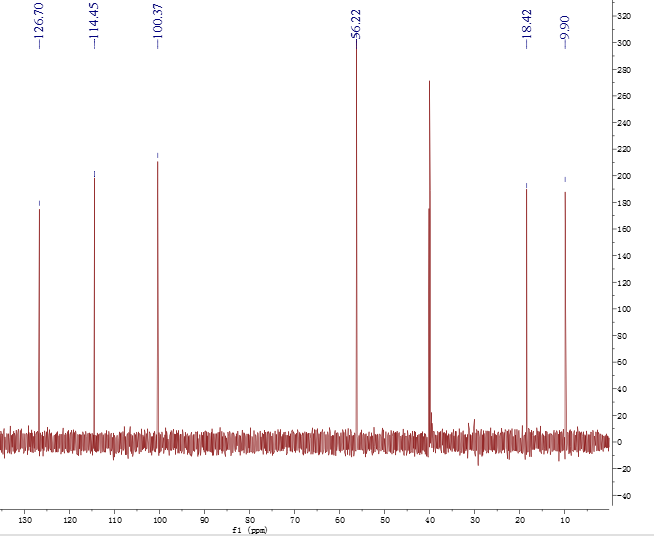


**Figure S22**. DEPT (150 MHz, DMSO-*d*_6_) spectrum of compound **3**
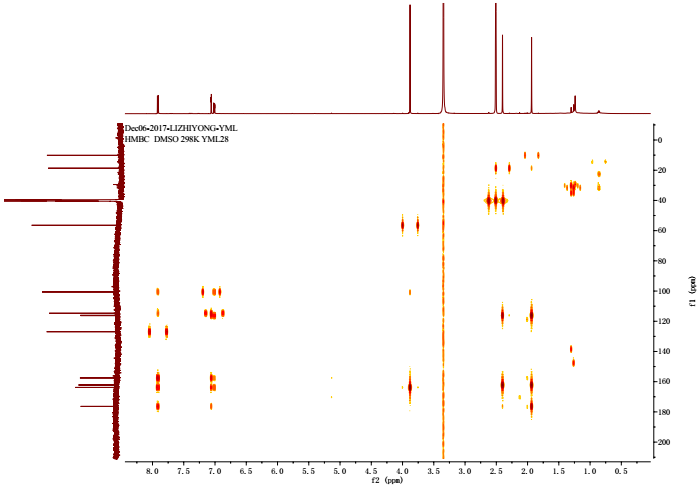


**Figure S23**. HMBC (DMSO-*d*_6_) spectrum of compound **3**


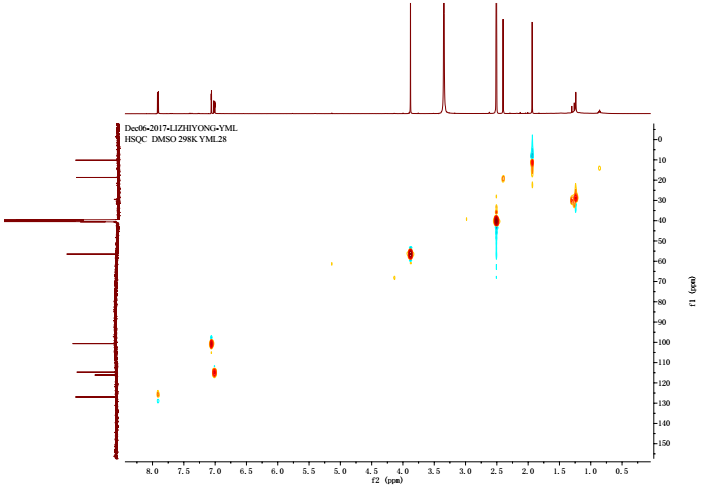


**Figure S24**. HSQC (DMSO-*d*_6_) spectrum of compound **3**


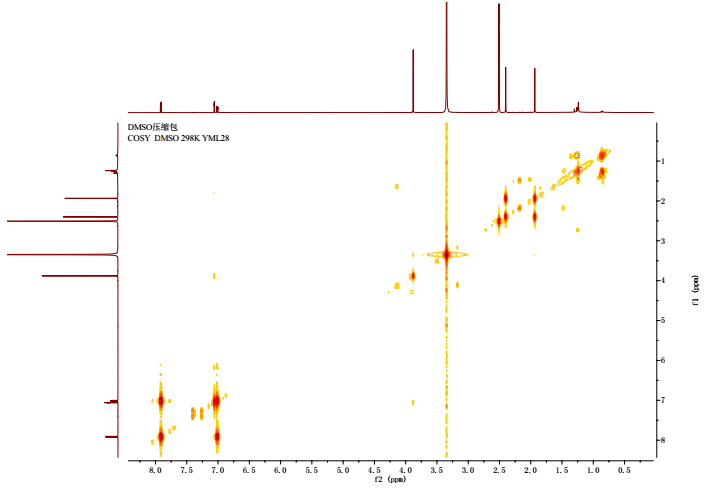


**Figure S25**. ^1^H–^1^H COSY (DMSO-*d*_6_) spectrum of compound **3**

**New compound statistics**

**Borrelidin J (1)*:*** a light yellow oil; [α]^25^_D_ –25.5 (*c* 4.0, MeOH); UV (*λ*_max_, MeOH) log *ε* 257 (2.6) nm; IR (KBr) *ν*_max_ 3375, 3035, 2957, 2921, 2874, 2210, 1958, 1715, 1637, 1589, 1433, 1409, 1380, 1308, 1196, 1021, 952, 785, 705 cm^–1^; ^1^H NMR (pyridine-*d*_5_, 600 MHz) 6.95 (1H, d, *J* = 11.0 Hz), 6.85 (1H, m), 6.48 (1H, m), 4.71 (1H, d, *J* = 8.0 Hz), 4.54 (1H, m), 3.83 (1H, m), 3.23 (1H, m), 2.93 (1H, m), 2.82 (1H, d, *J* = 13.5 Hz), 2.75 (1H, m), 2.63 (1H, m), 2.55 (1H, m), 2.21 (1H, m), 2.16 (1H, m), 2.05 (1H, m), 1.91 (1H, m), 1.84 (1H, m), 1.80 (1H, m), 1.75 (2H, m), 1.62 (2H, m), 1.48 (1H, m), 1.37 (1H, m), 1.27 (4H, m), 1.23 (1H, m), 1.15 (1H, m), 1.11 (3H, d, *J* = 6.5 Hz), 0.95 (1H, m), 0.93 (3H, d, *J* = 5.5 Hz), 0.92 (3H, d, *J* = 5.5 Hz); ^13^C NMR (pyridine-*d*_5_, 150 MHz), 179.4 (C-23), 175.4 (C-1), 144.1 (C-13), 143.3 (C-15), 126.4 (C-14), 120.4 (C-28), 117.2 (C-12), 74.0 (C-17), 71.5 (C-11), 70.7 (C-3), 49.7 (C-18), 47.2 (C-22), 45.9 (C-7), 41.5 (C-5), 41.2 (C-2), 40.5 (C-16), 39.7 (C-9), 36.5 (C-4), 35.8 (C-10), 31.7 (C-21), 30.3 (C-19), 27.3 (C-6), 27.2 (C-8), 26.1 (C-20), 20.9 (C-26), 20.0 (C-25), 15.4 (C-27), 14.7 (C-24); HRESIMS [M - H]^–^ *m/z* 506.3117 (C_28_H_44_NO_7_^–^, calcd. 506.3114).

**Borrelidin K (2)*:*** a light yellow oil; [α]^25^_D_ –14.0 (*c* 2.5, MeOH); UV (*λ*_max_, MeOH) log *ε* 257 (2.6) nm; IR (KBr) *ν*_max_ 3387, 3038, 2956, 2924, 2873, 2210, 1959, 1723, 1636, 1583, 1435, 1408, 1379, 1305, 1198, 1024, 973, 784, 706 cm^–1^; ^1^H NMR (pyridine-*d*_5_, 600 MHz) 6.96 (1H, d, *J* = 11.0 Hz), 6.84 (1H, m), 6.42 (1H, m), 4.71 (1H, d, *J* = 8.0 Hz), 4.55 (1H, m), 3.72 (1H, m), 3.64 (3H, s), 3.05 (1H, dd, *J* = 8.0, 8.0 Hz), 2.94 (1H, m), 2.82 (1H, m), 2.62 (1H, m), 2.53 (1H, m), 2.45 (1H, m), 2.22 (1H, m), 1.92 (2H, m), 1.87 (1H, m), 1.78 (1H, m), 1.76 (1H, m), 1.75 (1H, m), 1.62 (2H, m), 1.42 (1H, m), 1.37 (1H, m), 1.28 (4H, m), 1.25 (1H, m), 1.23 (1H, m), 1.16 (1H, m), 1.11 (3H, d, *J* = 6.5 Hz), 0.96 (1H, m), 0.94 (3H, d, *J* = 6.0 Hz), 0.93 (3H, d, *J* = 6.0 Hz); ^13^C NMR (pyridine-*d*_5_, 150 MHz), 177.3 (C-23), 175.3 (C-1), 144.0 (C-13), 143.0 (C-15), 126.5 (C-14), 120.3 (C-12), 117.3 (C-23), 73.7 (C-17), 71.5 (C-11), 70.7 (C-3), 51.3 (C-24), 50.3 (C-18), 46.8 (C-22), 45.9 (C-7), 41.5 (C-5), 41.1 (C-2), 40.5 (C-16), 39.8 (C-9), 36.5 (C-4), 35.8 (C-10), 31.7 (C-21), 29.9 (C-19), 27.3 (C-6), 27.2 (C-8), 25.8 (C-20), 20.9 (C-26), 20.0 (C-27), 15.3 (C-28), 14.7 (C-25); HRESIMS [M - H]^–^ *m/z* 520.3264 (C_29_H_46_NO_7_^–^, calcd. 520.3261).

**7-Methoxy-2,3-dimethylchromone-4-one (3)*:*** a brown yellow solid; UV (*λ*_max_, MeOH) log *ε* 224 (4.3), 291 (3.3) nm; IR (KBr) *ν*_max_ 3049, 3007, 2955, 2925, 2851, 2357, 1969, 1708, 1634, 1604, 1500, 1440, 1403, 1351, 1276, 1242, 1202, 1181, 1110, 1029, 930, 855, 822, 779, 765, 688; ^1^H NMR (DMSO, 600MHz) 7.91 (1H, d, J = 8.9 Hz), 7.06 (1H, d, J = 2.4 Hz), 7.01 (1H, dd, 8.9, J = 2.4 Hz), 3.88 (3H, s), 2.40 (3H, s), 1.93 (3H, s); ^13^C NMR (DMSO, 150 MHz), 176.4 (C-1), 163.8 (C-7), 162.2 (C-2), 157.6 (C-10), 127.0 (C-9), 116.3 (C-5), 116.1 (C-3), 114.7 (C-8), 100.6 (C-6), 56.5 (C-13), 18.7 (C-12), 10.2 (C-11); HRESIMS [M + H]^+^ *m/z* 205.0885 (C_12_H_13_O_3_^+^, calcd. 205.0888).
